# Heterogeneous DNA Methylation Patterns in the *GSTP1* Promoter Lead to Discordant Results between Assay Technologies and Impede Its Implementation as Epigenetic Biomarkers in Breast Cancer

**DOI:** 10.3390/genes6030878

**Published:** 2015-09-17

**Authors:** Grethe I. Grenaker Alnaes, Jo Anders Ronneberg, Vessela N. Kristensen, Jörg Tost

**Affiliations:** 1 Department of Cancer genetics, Institute for Cancer Research, Oslo University Hospital, Radiumhospitalet, Oslo 0310, Norway; E-Mails: Grethe.I.Grenaker.Alnas@rr-research.no (G.I.G.A.); Jo.Anders.Ronneberg@gmail.com (J.A.R.); v.n.kristensen@medisin.uio.no (V.N.K.); 2 Institute of Clinical Medicine, University of Oslo, Oslo 0318, Norway; 3 Department of Clinical Molecular Biology (EpiGen), University of Oslo, Ahus, 1478 Lørenskog, Norway; 4 Laboratory for Epigenetics and Environment, Centre National de Génotypage, CEA-Institut de Génomique, Evry 91000, France

**Keywords:** DNA methylation, breast cancer, *GSTP1*, biomarker, heterogeneity, method, MethyLight, MSP, pyrosequencing, mass spectrometry

## Abstract

Altered DNA methylation patterns are found in many diseases, particularly in cancer, where the analysis of DNA methylation holds the promise to provide diagnostic, prognostic and predictive information of great clinical value. Methylation of the promoter-associated CpG island of *GSTP1* occurs in many hormone-sensitive cancers, has been shown to be a biomarker for the early detection of cancerous lesions and has been associated with important clinical parameters, such as survival and response to treatment. In the current manuscript, we assessed the performance of several widely-used sodium bisulfite conversion-dependent methods (methylation-specific PCR, MethyLight, pyrosequencing and MALDI mass-spectrometry) for the analysis of DNA methylation patterns in the *GSTP1* promoter. We observed large discordances between the results obtained by the different technologies. Cloning and sequencing of the investigated region resolved single-molecule DNA methylation patterns and identified heterogeneous DNA methylation patterns as the underlying cause of the differences. Heterogeneous DNA methylation patterns in the *GSTP1* promoter constitute a major obstacle to the implementation of DNA methylation-based analysis of *GSTP1* and might explain some of the contradictory findings in the analysis of the significance of *GSTP1* promoter methylation in breast cancer.

## 1. Introduction

The analysis of epigenetic modifications has gained great interest in recent years, as it has become clear that epigenetics plays a key role in normal development, as well as in disease [1]. DNA methylation-based biomarkers bear the promise to contain valuable information for the early diagnosis of cancer, prognosis and tumor classification and might assist in the prediction of response to therapy (pharmacoepigenomics) [2,3]. Examples of DNA methylation-based biomarkers that are advanced in their development, validation and commercial implementation include the plasma-based detection of *SEPT9* methylation for the early detection of colorectal adenocarcinoma [4,5], the analysis of methylation in the promoter region of *MGMT* for the prediction of the response to alkylating agents, such as temozolomide, in the chemotherapy of glioblastomas [6] and the detection of hypermethylation of the glutathione S-transferase gene (*GSTP1*) for the distinction between prostate cancer and benign lesions in patients with high PSA levels [7,8].

Glutathione S-transferases, GSTs, are important for the detoxification of electrophilic metabolites of carcinogens and reactive oxygen species [9], and genetic and epigenetic variation particularly in *GSTP1* has been the focus of much research in different cancers [10]. Elevated expression of the *GSTP1* gene or protein has been reported to correlate with drug resistance in human cancers [11,12,13], and high levels have been associated with poor prognosis in breast and colon cancer [14,15]. Hypermethylation of the *GSTP1* promoter has been associated with gene silencing in multiple cancers [10,16]. In breast cancer, *GSTP1* CpG island hypermethylation has been found to be significantly associated with tumor size, lymph node metastasis and relapse-free survival [17,18]. In our previous studies, we showed that in a cohort of locally-advanced primary breast tumors treated with an anthracycline-based monotherapy, the methylation status of *GSTP1* was positively associated with overall survival and independent of other known clinical and histopathological parameters [19]. The DNA methylation susceptibility of the locus was regulated by an interplay of tumor characteristics, notably the estrogen receptor status, and the genetic background/haplotype of the locus [20]. DNA methylation at this locus was already present in pre-neoplastic lesions, such as ductal carcinoma *in situ* [21], and increased with tumor progression [22]. Methylation at the *GSTP1* promoter could thus be a target of choice for the detection of lesions with malignant potential complementing the results of mammography, as well as potentially for the stratification of patients for personalized treatment.

**Figure 1:**
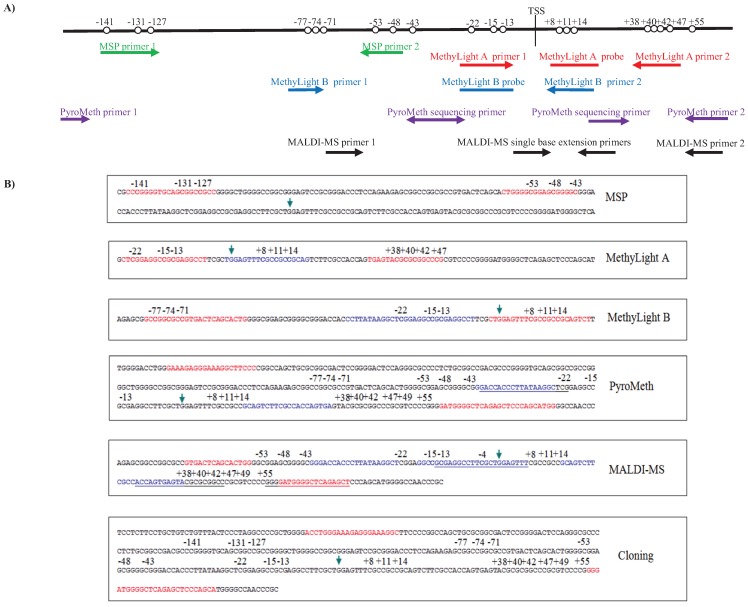
(**A**) Schematic view of the location of the MSP, MethyLight, PyroMeth and MALDI-MS assays relative to the CpG positions analyzed; (**B**) the location of the MSP, MethyLight, PyroMeth and MALDI-MS assays and the cloned PCR fragment in the *GSTP1* genomic sequence. The arrow indicates the transcription start site. PCR primers are shown in red. Probes, sequencing primers and mini-sequencing primers are shown in blue. CpGs in primers/probes are in bold.

Because of the potential added clinical value in the assessment of the DNA methylation status of *GSTP1*, we evaluated several widely-used sodium bisulfite conversion-dependent technologies to assess routinely the DNA methylation status of the *GSTP1* promoter in primary breast tumors and potentially other cancers (Figure 1): (1) methylation-specific PCR (MSP) [23]; (2) MethyLight [24]; (3) pyrosequencing (PyroMeth) [25]; and (4) MALDI mass-spectrometry (MALDI-MS) [20,26]. Due to the unexpected heterogeneity of the results of the different assays, we also cloned and sequenced the region −223 to +81 relative to the transcription start site of *GSTP1*, which allowed us also to resolve DNA methylation patterns of single molecules.

We discovered large differences in the performance of the different technologies, and the identified heterogeneous DNA methylation patterns might explain some of the contradictory findings in the analysis of the significance of *GSTP1* promoter methylation in breast cancer.

## 2. Experimental Section

### 2.1. Patient Material

Eighty-eight primary randomly-selected breast cancer tumors were analyzed for the DNA methylation status in the promoter region of *GSTP1*. DNA was isolated from snap frozen tumor tissue using phenol/chloroform extraction. DNA concentrations were determined using the Quant-iT™ dsDNA broad range assay kit (Invitrogen, Carlsbad, CA, USA) and normalized to a concentration of 50 ng/μL. As we have previously shown that the region analyzed by the different assays is unmethylated in normal breast tissue [19,20,22,27], we did not include normal breast epithelium in the current study.

### 2.2. Bisulfite Conversion

Bisulfite conversion of 1 µg of genomic DNA was carried out using the EpiTect Bisulfite kit (Qiagen, Hilden, Germany) according to the manufacturer’s instructions, and the bisulfite-treated DNA was eluted in 40 μL of the provided elution buffer.

### 2.3. Methylation Specific PCR, MSP

The two sets of MSP primers complementary to the unmethylated and methylated sequences after bisulfite treatment of genomic DNA have previously been published [28] (Supplementary Table S1). They target a 241-bp fragment in the *GSTP1* promoter, −145 to −43 relative to the transcription start site (TSS) (Figure 1). Each of the PCR primers covers more than one CpG, and the resulting PCR products are thus methylated in all or none of these CpGs. The PCR reactions were carried out using 25 ng of bisulfite-treated DNA, 2 U HotStarTaq DNA Polymerase (Qiagen), 1× HotStarTaq PCR buffer supplemented with 1.6 mM MgCl_2_ (Qiagen), 125 μM dNTPs and 5 pmol of forward and reverse PCR primer (Eurofins MWG Operon, Ebersberg, Germany) (Supplementary Table S1) in a total volume of 25 μL. The PCR program consisted of a denaturing step of 15 min at 95 °C followed by 40 cycles of 30 s at 95 °C, 30 s at 59 °C and 30 s at 72 °C, with a final extension of 7 min at 72 °C. The results were analyzed by 2% agarose gel electrophoresis.

### 2.4. Quantitative Methylation-Specific PCR, MethyLight

Two partly-overlapping MethyLight assays, MethyLight A and MethyLight B (Figure 1 and Supplementary Table S1), were used in separate real-time PCR-reactions. MethyLight A has previously been used at the NIH (Karen Woodson, personal communication), and MethyLight B has previously been published [29]. An endogenous control targeting Alu elements (ALU-C4, Supplementary Table S1) was used as the template input control [30]. All MethyLight reactions were carried out using 25 ng of bisulfite-treated DNA, 1× TaqMan^®^ Universal PCR Master Mix No AmpErase^®^ UNG (Applied Biosystems, Foster City, CA, USA), 3 pmol of forward and reverse PCR primer (Applied Biosystems) and 3 pmol 6'-carboxyfluorescein (FAM) labeled TaqMan MGB probe (Applied Biosystems) in a total volume of 10 μL in a 7900HT Fast Real-Time PCR System equipped with a 384-well plate module. The PCR program consisted of a denaturing step of 10 min at 95 °C followed by 40 cycles of 15 s at 95 °C and 60 s at 60 °C. Each sample DNA and a dilution series of commercially available bisulfite-treated methylated DNA (Qiagen) were run in triplicate. The average Ct-value was calculated from the triplicates, and Ct-values above 35 were excluded from the analysis. The copy number of methylated DNA in each methylated control DNA PCR reaction was calculated using the equation copy number = (ng DNA input × 6.022 × 10^23^)/(length (bp) of PCR-product 1 × 10^9^ × 660). Values were plotted against the Ct-values of the corresponding PCR reactions, and this standard curve was used to convert the Ct-values of the test samples into copy number values. The converted values of both the MethyLight A and B assays were normalized to the converted values of the endogenous control ALU-C4M, as previously described [30]. The percentage of methylated reference (PMR) value for each sample was calculated independently for both MethyLight assays using the equation PMR (percentage of methylated reference) = (converted and normalized value for test sample/converted and normalized value for 100% methylated DNA) × 100%.

### 2.5. Pyrosequencing

The DNA methylation patterns of 20 potentially methylated cytosines from −81 to +55 relative to the TSS (Figure 1) were analyzed by pyrosequencing according to Dejeux *et al.* [19]. None of the PCR primers covered any CpGs.

### 2.6. MALDI Mass-Spectrometry

The methylation status of the cytosines −22, +8 and +14, +38, +47 and +55 related to the TSS (Figure 1) was quantitatively analyzed by primer extension reaction followed by MALDI mass spectrometry-based read-out following the procedure described in detail in [26]. The extension primers spanning other CpGs were synthesized with C/T or A/G wildcards.

### 2.7. Cloning and Sequencing

The cloning and bisulfite sequencing of the promoter region was carried out for 8 samples. A 267-bp fragment, −223 to +81 relative to the TSS, was amplified using primers not covering any CpGs (Figure 1 and Supplementary Table S1). The PCR reactions were carried out using 20 ng of bisulfite-treated DNA, 2 U HotStar Taq DNA Polymerase (Qiagen), 1× HotStar Taq PCR buffer supplemented with 1.6 mM MgCl_2_ (Qiagen), 125 μM dNTPs and 5 pmol of forward and reverse PCR primers (Eurofins MWG Operon, Ebersberg, Germany) (Supplementary Table S1) in a total volume of 25 μL. The PCR program consisted of a denaturing step of 15 min at 95 °C followed by 35 cycles of 30 s at 95 °C, 30 s at 54 °C and 30 s at 72 °C, with a final extension of 7 min at 72 °C.

Three microliters of each PCR product were cloned into the pCR^®^2.1-TOPO TA vector (Invitrogen), transformed into chemically-competent DH5α™-T1^R^ *Escherichia coli* and grown overnight on LB plates containing 50 μg/mL ampicillin. Five to twelve colonies were picked from each LB plate and submerged in 50 μL H_2_O before denaturation for 5 min at 95 °C. The vector fragments containing the cloned PCR product were amplified using 3 μL DNA, 2 U HotStar Taq DNA Polymerase (Qiagen), 1× HotStar Taq PCR buffer supplemented with 1.6 mM MgCl_2_ (Qiagen), 125 μM dNTPs and 5 pmol M13 forward and reverse primer provided within the kit in a total volume of 25 μL. The PCR program consisted of a denaturing step of 15 min at 95 °C followed by 35 cycles of 30 s at 95 °C, 30 s at 54 °C and 30 s at 72 °C, with a final extension of 7 min at 72 °C.

The amplified vector fragments were analyzed by Sanger sequencing using BigDye^®^ Terminator v1.1 chemistry (Applied Biosystems). The sequencing reaction was carried out with 2 μL purified PCR product, 1× BigDye^®^ Terminator v1.1 Sequencing buffer, 2 μL Big Dye^®^ Terminator reaction mix v1.1 and 0.8 pmol M13 primer in a total volume of 10 μL. The sequencing reaction temperature profile consisted of a denaturing step of 2 min at 96 °C followed by 25 cycles of 15 s at 96 °C, 5 s at 50 °C and 4 min at 60 °C. The sequences were manually read after analysis with the Sequencing Analysis Software v5.3.1 (Applied Biosystems, Foster City, CA, USA).

### 2.8. RNA Expression

mRNA profiling were performed on 4 × 44 microarrays (Agilent, Santa Clara, CA, USA). Data were extracted and processed as previously described [31].

### 2.9. Statistical Analysis

Quantitative DNA methylation data provided by MethyLight, PyroMeth and MALDI-MS were compared using the non-parametric Spearman’s rank correlation coefficient. Data from the non-quantitative DNA methylation analysis method (MSP) were compared to the categorized DNA methylation data (unmethylated <5%) using Fisher’s exact test. DNA methylation and expression levels were compared using the non-parametric Spearman’s rank correlation coefficient. Survival analyses were performed using the Kaplan-Meier log rank test.

## 3. Results and Discussion 

### 3.1. Analysis of the DNA Methylation Status of the GSTP1 Promoter Using MSP and MethyLight

**Figure 2:**
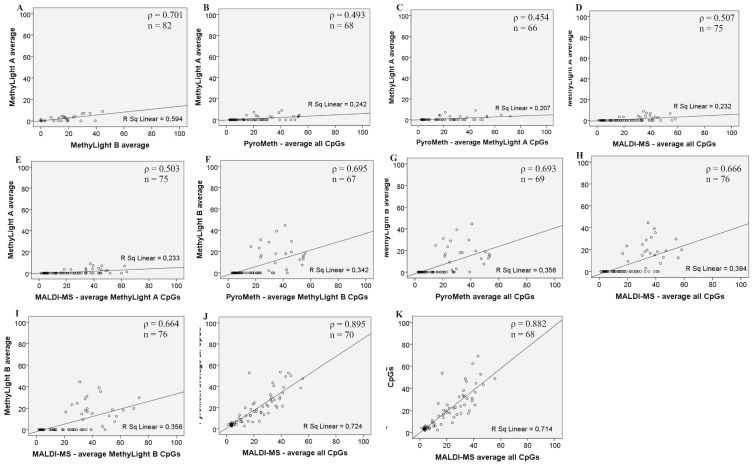
Scatterplots of the comparison of the two by two methylation analysis methods: (**A**) MethyLight A and MethyLight B; (**B**) MethyLight A and the average methylation % of all CpGs analyzed by PyroMeth; (**C**) MethyLight A and the PyroMeth average methylation % of the CpGs covered by the MethyLight A primers and probe; (**D**) MethyLight A and the average methylation % of all CpGs analyzed by MALDI-MS; (**E**) MethyLight A and the MALDI-MS average methylation % of the CpGs covered by the MethyLight primers and probe; (**F**) MethyLight B and the PyroMeth average methylation % of the CpGs covered by the MethyLight B primers and probe; (**G**) MethyLight B and the average methylation % of all CpGs analyzed by PyroMeth; (**H**) MethyLight B and the average methylation % of all CpGs analyzed by MALDI-MS; (**I**) MethyLight B and the MALDI-MS average methylation % of the CpGs covered by the MethyLight B primers and probe; (**J**) the average methylation % of all CpGs analyzed by PyroMeth and the average methylation % of all CpGs analyzed by MALDI-MS; (**K**) average methylation % of the same CpGs analyzed by PyroMeth and MALDI-MS (−22, +8, +14, +38, +47 and +55).

Due to their ease of use, we initially focused on the development of methylation-specific PCRs that have been widely used in the literature for the assessment of the methylation status of *GSTP1*. We analyzed the region spanning the transcription start of *GSTP1* using conventional, gel-based methylation-specific PCR (MSP), as well as the quantitative MethyLight assay. For the latter, the methylation status of Alu-repeats was used for the calibration and determination of the percentage of the methylated reference (PMR) value. We obtained information on the methylation status by MSP for 74 samples, while the MethyLight A primer/probe set provided the methylation information for 82 samples (Supplementary Table S2). However, when calculating the PMR values, 80% of the samples analyzed by the MethyLight A assay had undetermined or Ct values above 35, which would indicate the absence of DNA methylation at the *GSTP1* promoter. As the frequency of DNA methylation was surprisingly low compared to our previous studies in breast cancer and ductal carcinoma *in situ* (DCIS) [19,20,21] and to rule out any problem with the design of the assays, we analyzed the target region using another previously-published MethyLight assay [29]. However, this MethyLight B assay did detect DNA methylation in only 28% of the analyzed samples (Supplementary Table S2). Using the quantitative DNA methylation values, the results from the two MethyLight assays did correlate significantly, but only at ρ = 0.701 (Figure 2).

As the conventional MSP assay yields only qualitative information about the methylation status, the presence of PCR products obtained with the primers targeting the unmethylated and the methylated DNA template was compared to the categorized data (>5% methylated) from the quantitative MethyLight assays (Table 1).

**Table 1:** Fisher’s exact test on the MSP analysis and the categorized results from the quantitative methods MethyLight A and B, PyroMeth and MALDI-MS.

MethyLight A ^1^										
MethyLight B ^2^	0.0003									
	(*n* = 80)									
PyroMeth-MethyLight A CpGs ^3^	4.0097 × 10^−15^ (*n* = 67)									
PyroMeth-MethyLight B CpGs ^4^		2.8522 × 10^−11^ (*n* = 67)								
PyroMeth - MALDI-MS CpGs ^5^										
PyroMeth-average of the analyzed CpGs ^6^	2.0858 × 10^−16^ (*n* = 66)	5.3771 × 10^−7^ (*n* = 69)								
MALDI-MS-MethyLight A CpGs ^7^	5.4416 × 10^−18^ (*n* = 73)		0.4639							
			(*n* = 70)							
MALDI-MS-MethyLight B CpGs ^8^		1.8476 × 10^−6^ (*n* = 76)		0.0929						
				(*n* = 68)						
MALDI-MS-average of the analyzed CpGs ^9^	2.1717 × 10^−17^ (*n* = 73)	1.3602 × 10^−7^ (*n* = 76)			0.5727	0.8495				
					(*n* = 68)	(*n* = 70)				
MSP ^1^	0.0011	0.9999				2.5759 × 10^−6^			7.7596 × 10^−7^	
	(*n* = 71)	(*n* = 73)				(*n* = 63)			(*n* = 71)	
	MethyLight A ^1^	MethyLight B ^2^	PyroMeth-MethyLight A CpGs ^3^	PyroMeth-MethyLight B CpGs ^4^	PyroMeth-MALDI-MS CpGs ^5^	PyroMeth-average of the analyzed CpGs ^6^	MALDI-MS-MethyLight A CpGs ^7^	MALDI-MS-MethyLight B CpGs ^8^	MALDI-MS-average of the analyzed CpGs ^9^	MSP ^10^

The Fisher’s exact test on the results from the MSP analysis show good correlation with the categorized data (unmethylated <5%) from the MethyLight B analysis (*p* = 0.9999), while the other quantitative methods MethyLight A, PyroMeth and MALDI-MS show low correlation to the MSP results.^1^ MethyLight A CpG positions (pos.): −22, −15, −13, +8, +11, +14, +38, +40, +42 and +47; ^2^ MethyLight B CpG pos.: −77, −74, −71, −22, −15, −13, +8, +11 and +14; ^3^ PyroMeth average of CpG pos. covered by the MethyLight A primers and probe: −22, −15, −13, +8, +11, +14, +38, +40, +42 and +47; ^4^ PyroMeth average of CpG pos. covered by the MethyLight B primers and probe: −77, −74, −71, −22, −15, −13, +8, +11 and +14; ^5^ PyroMeth average of CpG pos. also analyzed by MALDI-MS: −22, +8, +14, +38, +47 and +55; ^6^ PyroMeth average of all CpGs analyzed: pos. −77, −74, −71, −48, −43, −22, −15, −13, +8, +11, +14, +38, +40, +42, +47 and +55; ^7^ MALDI-MS average of CpG pos. covered by the MethyLight A primers and probe: −22, −15, −13, +8, +11, +14, +38, +40, +42 and +47; ^8^ MALDI-MS average of CpG pos. covered by the MethyLight B primers and probe: −77, −74, −71, −22, −15, −13, +8, +11 and +14; ^9^ MALDI-MS average of all CpGs analyzed: pos. −22, +8 and +14, +38, +47 and +55; ^10^ MSP primers for genomic DNA and bisulfite converted DNA covering CpG pos. −141, −131, −127, −53 and −48.

The results from the MethyLight B method showed a high correlation with the MSP analysis (*p* = 0.99; *n* = 73), while the MethyLight A was significantly different from the MSP results (*p* = 0.0011; *n* = 71).

Similarly, the categorized results from the MethyLight A and MethyLight B assays were significantly different (*p* = 0.0003; *n* = 80; Table 1), indicating that different samples were called methylated by both assays and suggesting technical difficulties for assessing the methylation status. Increasing the amount of DNA template in the real-time PCR reaction did not yield an increased detection ratio for the MethyLight assays, but confirmed the observed results (Supplementary Figure S1).

### 3.2. Analysis of the GSTP1 Promoter by Pyrosequencing and MALDI-MS-Based Epigenotyping

As the low detection rate of *GSTP1* methylation could be due to differences in the methylation frequencies between collections, we analyzed the same samples using two quantitative methods: pyrosequencing and MALDI-MS-based epigenotyping. Using a cut-off of 5%, significant DNA methylation levels were detected for 72% of the samples by pyrosequencing, as well as by mass spectrometry. Taking only the positions into account that were covered by the MethyLight assays, between 68.6% and 85.7% of the samples displayed significant methylation levels, indicating that a large proportion of methylated samples was missed by the MethyLight and MSP assays.

As the MALDI-MS-based assay analyzed only five specific CpGs, the average percentage of methylated cytosines in position −22, +8, +14, +38 and +47 from the PyroMeth and MALDI-MS analysis was calculated for each of the methods. Spearman’s correlation coefficient demonstrated that the results from PyroMeth and MALDI-MS correlate at the *p* = 0.01 level with a ρ = 0.882 (*n* = 68) (Table 2). The scatter plots, comparing the correlation between the results obtained from the different quantitative technologies (Figure 2), further confirmed the tight correlation between the PyroMeth and MALDI-MS results, providing further evidence for a problem in the MSP-based assays. We then compared the DNA methylation levels for the CpGs covered by the MethyLight assays to the average of the exact same CpGs analyzed by the quantitative methods. The amplification primers used in the MethyLight A assay cover the CpGs in positions −77, −74 and −71 (forward amplification primer), −22, −15 and −13 (probe) and +8, +11 and +14 (reverse amplification primer) (Figure 1). The MethyLight A and PyroMeth correlated at the 0.01 level with a ρ = 0.454 (*n* = 66) (Table 2). For the MethyLight B assay, covering positions −22, −15 and −13 (forward amplification primer), +8, +11 and +14 (probe) and +38, +40, +42 and +47 (reverse amplification primer) (Figure 1), a Spearman’s correlation coefficient of ρ = 0.695 was obtained (*n* = 67) (Table 2). Comparison of the categorized (>5% methylated) methylation data obtained by PyroMeth and MALDI-MS with the MSP results showed significant differences between the quantitative methods and MSP (PyroMeth *vs.* MSP *p* = 2.58 × 10^−^^6^ (*n* = 63); MALDI-MS *vs.* MSP *p* = 7.76 × 10^−^^7^ (*n* = 71), Table 1), while PyroMeth and MALDI-MS data were essentially identical (R^2^ = 0.895; *p* = 0.8495). Comparing the categorized results from the quantitative methods to the data from the MethyLight analyses confirmed the significant differences (MethyLight A *vs.* PyroMeth: *p* = 4.01 × 10^−^^15^ (*n* = 67); MethyLight B *vs.* PyroMeth: *p* = 2.85 × 10^−^^11^ (*n* = 67); MethyLight A *vs.* MALDI-MS: *p* = 2.09 × 10^−^^16^ (*n* = 66); MethyLight B *vs.* MALDI-MS: *p* = 1.85 × 10^−^^6^ (*n* = 76); Table 1).

**Table 2:** The quantitative methylation analysis methods, MethyLight, PyroMeth and MALDI-MS, were compared using Spearman’s correlation coefficient, ρ. Comparison of the methylation percentage of the same CpG positions (−22, +8, +14, +38, +47 and +55) from the PyroMeth and MALDI-MS methods showed the highest correlation, ρ = 0.882 (*n* = 68). The MethyLight A method showed the overall lowest correlation to the other methods. PMR, percentage of the methylated reference.

Assay	ρ N	MethyLight A Average PMR	MethyLight B Average PMR	PyroMeth Average % of All Analyzed CpGs	PyroMeth Average % of MethyLight A CpGs	PyroMeth Average % of MethyLight B CpGs	PyroMeth Average % of MALDI-MS CpGs	MALDI-MS Average % of All Analyzed CpGs	MALDI-MS Average % of MethyLight A CpGs	MALDI-MS Average % of MethyLight B CpGs	MALDI-MS Average % of PyroMeth CpGs
MethyLight A average PMR	ρ	1.000	0.071	0.493	0.454	0.480	0.445	0.507	0.503	0.482	0.507
	N	82	82	68	66	66	66	75	75	75	75
MethyLight B average PMR	ρ	0.701	1.000	0.693	0.646	0.695	0.634	0.666	0.675	0.664	0.666
	N	82	85	69	67	67	67	76	76	76	76
PyroMeth average % of all analyzed CpGs	ρ	0.493	0.693	1.000	0.982	0.991	0.983	0.895	0.891	0.897	0.895
	N	68	69	72	70	70	70	70	70	70	70
PyroMeth average % of MethyLight A CpGs	ρ	0.454	0.646	0.982	1.000	0.967	0.993	0.886	0.880	0.893	0.886
	N	66	67	70	70	68	70	68	68	68	68
PyroMeth average % of MethyLight B CpGs	ρ	0.480	0.695	0.991	0.967	1.000	0.966	0.902	0.900	0.911	0.902
	N	66	67	70	68	70	68	68	68	68	68
PyroMeth average % of MALDI-MS CpGs	ρ	0.445	0.634	0.983	0.993	0.966	1.000	0.882	0.876	0.882	0.882
	N	66	67	70	70	68	70	68	68	68	68
MALDI-MS average % of all analyzed CpGs	ρ	0.507	0.666	0.895	0.886	0.902	0.882	1.000	0.997	0.984	1.000
	N	75	76	70	68	68	68	79	79	79	79
MALDI-MS average % of MethyLight A CpGs	ρ	0.503	0.375	0.891	0.880	0.900	0.876	0.997	1.000	0.987	0.997
	N	75	76	70	68	68	68	79	79	79	79
MALDI-MS average % of MethyLight B CpGs	ρ	0.482	0.664	0.897	0.893	0.911	0.882	0.984	0.987	1.000	0.984
	N	75	76	70	68	68	68	79	79	79	79
MALDI-MS average % of PyroMeth CpGs	ρ	0.507	0.666	0.895	0.886	0.902	0.882	1.000	0.997	0.984	1.000
	N	75	76	70	68	68	68	79	79	79	79

ρ: Spearman’s correlation coefficient; N: Number of samples with data using both assays used in the comparison. CpGs covered by the MethyLight A primers and TaqMan probe: positions (pos.) −22, −15, −13, +8, +11, +14, +38, +40, +42 and +47; CpGs covered by the MethyLight B primers and TaqMan probe: pos. −77, −74, −71, −22, −15, −13, +8, +11 and +14; PyroMeth average % of all analyzed CpGs: pos. −77, −74, −71, −48, −43, −22, −15, −13, +8, +11, +14, +38, +40, +42, +47 and +55; PyroMeth average % of CpGs covered by the MethyLight A primers and probe: pos. −22, −15, −13, +8, +11, +14, +38, +40, +42 and +47; PyroMeth average % of CpGs covered by the MethyLight B primers and probe: pos. −77, −74, −71, −22, −15, −13, +8, +11 and +14; PyroMeth average % of CpGs analyzed by MALDI-MS: pos. −22, +8, +14, +38, +47 and +55; MALDI-MS average % of CpGs covered by the MethyLight A primers and probe: pos. −22, −15, −13, +8, +11, +14, +38, +40, +42 and +47; MALDI-MS average % of CpGs covered by the MethyLight B primers and probe: pos. −77, −74, −71, −22, −15, −13, +8, +11 and +14; MALDI-MS average % of all analyzed CpGs: pos. −22, +8, +14, +38, +47 and +55.

**Figure 3:**
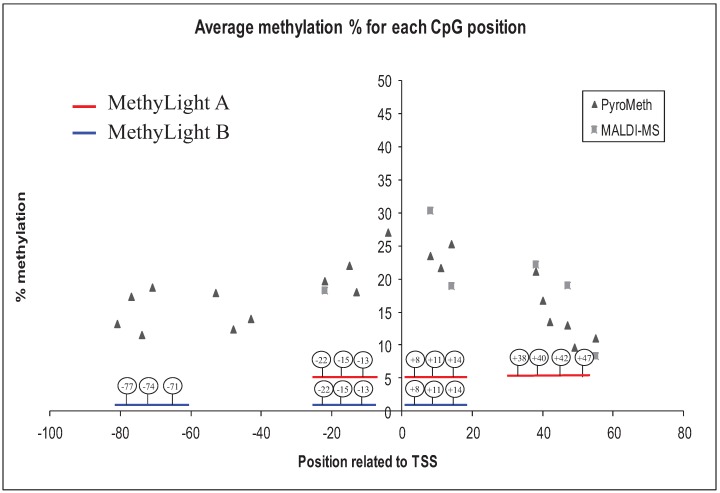
The average methylation percentage for each CpG position analyzed by PyroMeth and MALDI-MS. The positions of the MethyLight A and MethyLight B primers and probes are shown with red and blue lines, respectively. TSS, transcription start site.

Both MethyLight assays are spanning the transcription start site of *GSTP1,* which is the most commonly-analyzed region. The analysis of the very same region by PyroMeth/MALDI-MS did demonstrate an increase of the DNA methylation levels around the TSS and the subsequent decrease in the downstream region following the TSS (Figure 3), indicating that the most aberrantly methylated region was analyzed.

In contrast to the quantitative methods that rely on methylation-independent amplification (no potentially methylated cytosines are included in the amplification primers), MethyLight and MSP rely on methylated cytosines for efficient amplification after bisulfite treatment. We therefore hypothesized that the decrease of DNA methylation might interfere with the efficient primer binding of the amplification primers used for the MSP assays. This should particularly concern the MethyLight A assay, which had a more downstream binding site for the reverse amplification primer, as well as the MSP assay, which had the binding site for the forward amplification primer at CpGs 140 bases upstream of the TSS, but only to a smaller extent for the MethyLight B assay.

### 3.3. Bisulfite Sequencing of Single Molecules Reveals the Unexpected Presence of Heterogeneous DNA Methylation Patterns

**Figure 4:**
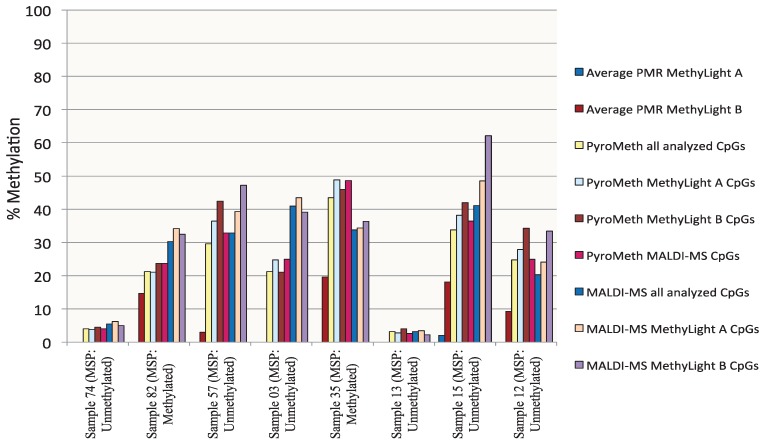
The percentage of DNA methylation of eight cloned and sequenced samples measured by MSP, MethyLight A, MethyLight B, PyroMeth and MALDI-MS shows a variability of DNA methylation levels, both between the methods and in positions within the PyroMeth method.

**Figure 5:**
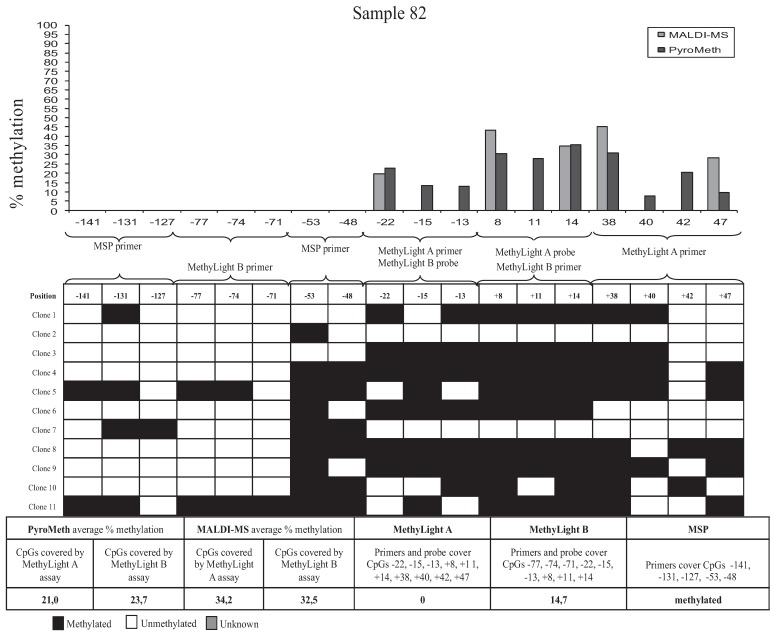
Results from cloning of a 267-bp PCR fragment of the *GSTP1* promoter. Sample 82 shows a different degree of methylation between each of the CpGs analyzed. CpG positions related to TSS analyzed by PyroMeth: −81, −77, −74, −71, −53, −48, −43, −22, −15, −13, −4, +8, +11, +14, +38, +40, +42, +47, +49; and +55 CpG positions related to TSS analyzed by MALDI-MS: −22, +8, +14, +38, +47 and +55.

**Figure 6:**
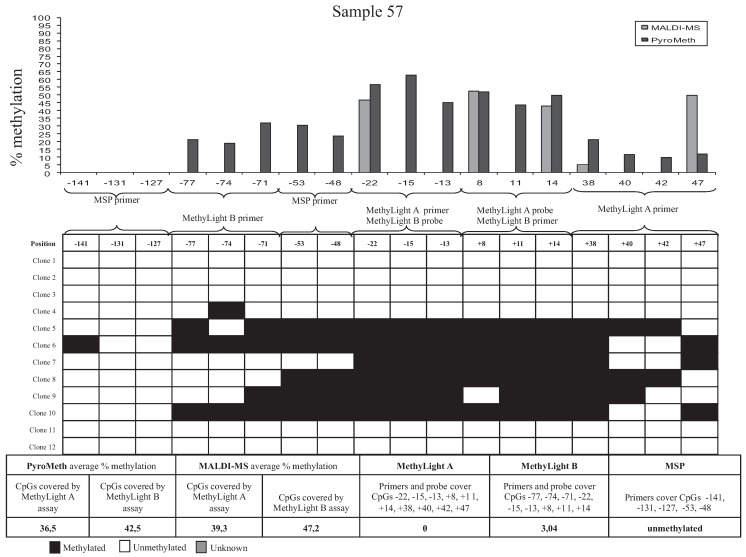
Results from cloning of a 267-bp PCR fragment of the *GSTP1* promoter. Sample 57 shows different degrees of methylation between each of the CpGs analyzed. CpG positions related to TSS analyzed by PyroMeth: −81, −77, −74, −71, −53, −48, −43, −22, −15, −13, −4, +8, +11, +14, +38, +40, +42, +47, +49; and +55 CpG positions related to TSS analyzed by MALDI-MS: −22, +8, +14, +38, +47 and +55.

To analyze the impact of DNA methylation patterns on the efficacy of the amplification in the MSP assays, we selected eight samples, which were analyzed by the “gold standard” technology of sequencing and cloning, which provides additional information by revealing molecule-specific DNA methylation patterns, including co-methylation patterns of specific CpGs on the same DNA molecule. We selected two samples that were found to be unmethylated by all technologies used (Samples 74 and 13, Figure 4), two samples found to be significantly methylated by PyroMeth and MALDI, but unmethylated by MSP and the MethyLight assays (Samples 57 and 03), two samples unmethylated by MethyLight A, but methylated by all other technologies, whereby the methylation level was underestimated to a varying degree in the MethyLight B assay (Samples 82 and 35), and two samples, unmethylated by MSP and MethyLight A, but methylated by all other technologies, whereby the methylation level was again underestimated in the MethyLight B assay (Samples 12 and 15). Sequencing of the cloned PCR fragments revealed that the DNA methylation patterns of the cytosines on the same strand were very heterogeneous. The results from the analysis of the different clones, as well as the methylation percentage obtained by the different methods are shown in Figure 5 for Sample 82, Figure 6 for Sample 57 and Supplementary Figure S2 (Samples 74, 13, 35, 15, 12 and 03). The two samples that were scored as unmethylated by all five technologies (Samples 74 and 13, Supplementary Figure S2A,B) only showed very sporadically methylated cytosines on single clones confirming the absence of DNA methylation in these two samples.

Sample 82 (Figure 5) displayed more extensive methylation than Sample 74, though not all CpGs on a cloned molecule were consistently methylated. Clone 5 had methylated cytosines at all CpGs, except positions −127 (covered by MSP primer), −71 (covered by MethyLight B primer), −22 (covered by MethyLight A primer and MethyLight B probe) and +42 (covered by MethyLight A primer) relative to the TSS. Clone 11 was also extensively methylated, except for the CpG’s −127 (covered by MSP primer), −22 and −13 (both covered by MethyLight A primer and MethyLight B probe) and +40 and +42 (both covered by MethyLight A primer), while Clone 2 had only one methylated CpG in position −53 (covered by MSP primer). The PyroMeth and MALDI-MS analysis of Sample 82 showed an average methylation percentage of 21.0 and 34.2 for the CpG positions covered by MethyLight A and 23.7 and 32.5 for the positions covered by MethyLight B. The MethyLight A assay scored this sample as unmethylated (0%) and the MethyLight B assay as 14.7% methylated. None of the sequenced clones were methylated in all CpG positions covered by the two MethyLight assays, which explains the underestimation of the DNA methylation degree for this sample. Similar heterogeneous DNA methylation patterns with clones rather, but not consistently, methylated were found for Sample 35 (Supplementary Figure S2C), which was the other sample found to be methylated by PyroMeth, MALDI-MS, MethyLight B and MSP, but not MethyLight A.

The PyroMeth and MALDI-MS analysis of this sample showed an average methylation percentage of 48.8 and 34.4 for the CpG positions covered by MethyLight A and 46.0 and 36.3 for the positions covered by MethyLight B. The methylation percentage obtained by MethyLight B (19.6%) was thus again largely underestimated.

Heterogeneous methylation patterns also explained the large underestimation of the methylation percentage by MethyLight B for Sample 15 (Supplementary Figure S2D), which showed less methylation at the CpGs upstream of position −71, which are covered by one of the MSP primers and one of the MethyLight B primers, leading to less efficient amplification. On the other hand, a low methylation level downstream of the TSS rendered amplification inefficient for Sample 12 (Supplementary Figure S2E), leading to significant underestimation of the methylation degree by MethyLight B. For Samples 57 (Figure 6) and 03 (Supplementary Figure S2F), only part of the clones contain methylated CpGs, though none of the clones had all CpG methylated that are covered by a primer or probe of an MSP or MethyLight assay. Therefore, the assays with primers not covering any CpGs (PyroMeth and MALDI-MS) detected this sample as methylated, while MethyLight A and B and MSP misinterpreted these samples as unmethylated.

### 3.4. Methylation Call Rates Have a Significant Impact on the Correlation to Expression Levels and Clinical Parameters

**Figure 7:**
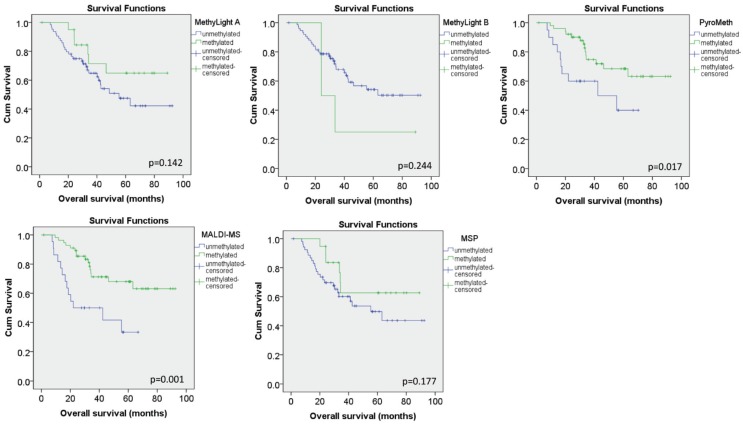
Impact of characterized DNA methylation levels on overall survival analysis. Kaplan Meier curves for the categorized DNA methylation data for the MethyLight A, MethyLight B, PyroMeth, MALDI-MS and MSP assays. P-values from the log-rank test are given within the respective panels.

We further investigated the impact of the results obtained by the different technologies on their correlation with the expression levels of the same tumors using Spearman correlation. No significant correlation was found for the MSP and the MethyLight A assay, while the MethyLight B assay showed a very moderate (ρ = −0.02), but significant inverse correlation (*p* = 0.026). The quantitative technologies however showed a strong and significant correlation (ρ = −0.480, *p* < 0.001 for MALDI and ρ = −0.486, *p* < 0.001 for PyroMeth) with the expression levels. Using categorized DNA methylation values, only 3/64 tumors (8%) were found to display a loss of expression while showing no detectable DNA methylation levels by the quantitative technologies, while this was the case for 29/64 (45%) samples for the MethyLight B assay and even more for the MSP and MethyLight A assay. In addition, methylation assessed by MALDI-MS and PyroMeth showed a significant impact on overall survival, while this association was not found for the MethyLight and MSP assays (Figure 7).

### 3.5. Discussion

Biomarkers capable of distinguishing cancerous cells from normal ones must be specific, sensitive and optimally detectable in specimens obtained through minimally-invasive procedures to be clinically applicable. The five methods compared within this study use bisulfite-treated DNA [32] as a template for PCR amplification, enabling quantitative analysis of the DNA methylation patterns by translating the epigenetic difference into easily measurable genetic polymorphisms. MSP, MethyLight, PyroMeth and MALDI-MS differ in accuracy, throughput, labor intensity and, most importantly, in the kind and amount of information gained.

MSP allows the amplification of virtually any CpG sites after bisulfite treatment with two pairs of primers for amplification, complementary to the methylated and the unmethylated sequences, respectively [23]. Primers hybridize to sequences with at least two methylation variable positions (CpGs) to obtain the necessary specificity for selective amplification, which enables the detection of one allele in the presence of a 1000-fold excess of the other [23]. The presence or absence of an amplification product analyzed on a conventional agarose gel reveals the methylation status. Despite its shortcomings, it is the most widely-used technology for DNA methylation analysis, as it does not require any expensive instrumentation and a large number of samples can be rapidly assessed.

MethyLight is based on the TaqMan^®^ assay principle: in addition to the two amplification primers, a third oligonucleotide, dually labeled with a fluorescent reporter and a quencher dye, hybridizes to a target sequence in the amplified region [24]. The addition of a third probe that has to anneal correctly to the DNA methylation patterns of the synthesized template improves sensitivity, as well as specificity compared to conventional MSP. The simple one-step procedure makes MethyLight a rapid high-throughput assay for quantitative DNA methylation analysis, providing information if molecules with a certain methylation pattern are present in the sample, like conventional MSP, but also reporting on how many of them there are.

PyroMeth is a real-time sequencing method where the nucleotides are added stepwise and a luminescence signal reflecting the number of nucleotides incorporated is generated [25]. Amplification is performed independent of the DNA methylation status of the target region, and results reflect the average DNA methylation degree among millions of molecules at a CpG of interest. This method yields highly quantitative information about the methylation status at single-CpG resolution within the length of the sequence read (~120 bp).

The GOOD assay for epigenotyping is a quantitative high-throughput method, which uses a mass spectrometry-based read-out of the signal intensity of single base primer extension products from multiplex extension reactions using an extension primers terminating immediately upstream of a CpG under investigation [26].

Methylation-specific PCR [23] and methylation-specific real-time PCR-based methods, such as MethyLight [24], HeavyMethyl [33] or Quantitative Analysis of Methylated Alleles (QAMA; [34]), have proven well suited for the detection of cancer-specific methylation patterns in primary tumor tissues, but also for the detection of very low levels of methylation in body fluids in contact with the tumor site and in circulating DNA [3]. These technologies are most commonly applied to validate and analyze the performance of DNA methylation-based biomarkers.

The DNA methylation patterns of the CpGs in the region from −223 to +81 relative to TSS of *GSTP1* are heterogeneous, as postulated from the variable DNA methylation degree of consecutive CpGs assessed by pyrosequencing or MALDI-MS and confirmed by the cloning and sequencing experiment. This heterogeneity leads to a high divergence between the methylation data generated by the two MethyLight assays and the PyroMeth/MALDI-MS analyses, while results correlated well between PyroMeth and MALDI-MS. Similar observations have been previously made for the promoter region of *ID4* in prostate cancer*,* which was analyzed by either gel-based MSP or pyrosequencing [35]. Significant differences could, as in our study, be attributed to partial and heterogeneous DNA methylation patterns at the underlying CpGs.

The discordant results are largely attributable to the differences in the assay design. The MethyLight probes will only gain the fluorescence signal if all cytosines covered by the primers and probes are consistently methylated in the genomic DNA, and the MethyLight assay designs will therefore give limited information on the methylation status. Both PyroMeth and MALDI-MS analyze each of the CpGs separately and provide quantitative information of the methylation level of individual CpGs with a quantitative resolution of better than 5% [25,26]. However, both technologies require the DNA methylation to be a frequent event, as their limit of detection is around 5% for the minor epi-allele and are not closed tube assays, requiring manual intervention and sample preparation after the amplification step, which is not desirable in a routine clinical setting.

The results of our study suggest that sequences containing CpGs with well-known DNA methylation patterns may be cursorily analyzed by MethyLight at a relatively high throughput. The heterogeneity of the DNA methylation patterns will increase the risk of false negative results exemplified by the large number of samples that were called unmethylated by the different MSP and MethyLight assays. The MSP/MethyLight primers used in this experiment cover more than one CpG position, as most published examples of MSP and MethyLight primers, and the consistency of the DNA methylation status between these closely-neighbored CpGs will affect the annealing efficiency of the primers to the template when the optimal PCR conditions are applied, which are in most cases developed on commercial or self-made completely methylated and unmethylated DNA templates that display in most cases homogeneous DNA methylation patterns.

With the exception of *MGMT*, *MLH1*, *p15* and *ID4*, which have been studied in some detail [35,36,37,38,39], relatively few studies have attempted to identify specific small regions in CpG islands or other regions of interest that correlate best with the phenotype or clinical parameter under analysis [40,41]. While heterogeneous DNA methylation patterns have been recognized for some time, the phenomenon is still largely neglected for the development of DNA methylation-based biomarkers [42], and although the results obtained with technologies using methylation dependent amplification will be invariably confounded by heterogeneous DNA methylation patterns, they are among the most widely-used technologies due their ease of use and available instrumentation.

The observed heterogeneous DNA methylation patterns and the re-use of published assays in different studies without re-evaluation of the performance of the assay in the samples of interest might also at least partly explain the contradictory findings for the role of *GSTP1* in breast cancer and the difficulties to reproduce the associations between *GSTP1* promoter hypermethylation and clinical and histopathological parameters.

Previous studies using MSP or MethyLight reported a frequency of hypermethylation of 13% to 30% for *GSTP1* in breast cancer [16,18,43,44,45,46], which is inferior to the frequency that we [19,20,21] and others [47,48] found using quantitative methods, such as pyrosequencing or MALDI mass spectrometry. Of note, in a large-scale study analyzing 839 breast cancer samples using the very same published MethyLight B assay, a methylation frequency similar to the one in our study was found (27.8% *vs.* 24.7%) [45], suggesting that a large proportion of methylated cases might have been missed due to the heterogeneous DNA methylation. As we show in this study (Figure 7), the lower detection rate of methylated samples leads to a loss of the prognostic significance of DNA methylation patterns in the *GSTP1* promoter, and this might also be true for the large-scale study.

This problem will be even more accentuated when analysis is performed in serum or plasma samples, where the detection poses a number of technological challenges due to the minute amounts of methylated molecules shed from the tumors into the bloodstream. In combination with the presence of heterogeneous DNA methylation patterns, the low quantity might preclude its use for population-wide screening using technologies based on the concomitant methylation of CpGs in close proximity, which will not have the required sensitivity. For example in a large case-control study analyzing *GSTP1* methylation in pre-diagnostic serum of breast cancer patients using the MethyLight B primer set, the detected DNA methylation patterns were unable to distinguish cases and controls with detection frequencies of DNA hypermethylation of below 10% [49]. The heterogeneous DNA methylation patterns revealed in our study in combination with the low prevalence of target molecules in the analyzed fluid could provide an explanation for this low detection rate.

## 4. Conclusions

Heterogeneous DNA methylation patterns will constitute a major obstacle to the development of reliable and reproducible assays and assessment of the clinical performance of DNA methylation-based biomarkers if not adequately controlled for. The recent advances in massively parallel sequencing, as well as single-molecule PCR provide the necessary molecule-specific DNA methylation patterns with little effort and enable one to investigate heterogeneous DNA methylation patterns at a large scale. Our study demonstrates that prior knowledge of the DNA methylation pattern of a regulatory region of interest is an important pre-requisite for the evaluation of DNA methylation-based biomarkers and might be equally important for well-accepted criteria, such as a well-defined and phenotyped study population and appropriate controls. There is increasing evidence that GSTs, and GSTP1 in particular, play an important role in drug resistance in breast and other cancers, and therefore, accurate and reliable assays determining the promoter methylation of this gene might be of great clinical value for patient stratification prior to treatment decision, as well as for the early detection of neoplasms.

## Supplementary Files

Supplementary File 1
