# Supplementary material for: Heterogeneous DNA Methylation Patterns in the GSTP1 Promoter Lead to Discordant Results between Assay Technologies and Impede Its Implementation as Epigenetic Biomarkers in Breast Cancer
Source: Genes (Basel). 2015 Sep 17;6(3):878–900. doi: 10.3390/genes6030878 (PMC4584334; doi:10.3390/genes6030878)
Supplement: Supplementary File 1 [file genes-06-00878-s001.pdf]

Supplementary Materials

Table S1. The oligonucleotides used in the MSP, MethyLight, PyroMeth and the MALDI-MS methylation analysis and the cloning of a region of the GSTP1 promoter.

Methylation method	Oligo name	Sequence 5'–3'	Size of PCR product (bp)	Position in GSTP1 promoter (related to transcription start site, TSS)
MSP	PCR primer 1, non-converted CpG's	TTCGGGGTGTAGCGGTCGTC	241	−143 to −43
	PCR primer 2, non-converted CpG's	GCCCCAATACTAAATCACGACG		
	PCR primer 1, converted CpG's	GATGTTTGGGGTGTAGTGGTTGTT	241	−143 to −43
	PCR primer 2, converted CpG's	CCACCCCAATACTAAATCACAACA		
MethyLight A	PCR primer 1	TTCGGAGGTTGCGAGGTTT	72	−24 to +48
	PCR primer 2	ACGAACCGCGCGTACTCA		
	TaqMan probe	6-FAM-TGGAGTTTCGTCGTCGTAG-MGBNFQ		−1 to +18
MethyLight B	PCR primer 1	GTCGGCGTCGTGATTTAGTATTG	100	−79 to +21
	PCR primer 2	AAACTACGACGACGAAACTCCAA		
	TaqMan probe	6FAM-AAACCTCGCGACCTCCGAACCTTATAAAA-MGBNFQ		−34 to −7
ALU-C4M endogenous control	PCR primer 1	GGTTAGGTATAGTGGTTTATATTTGTAATTT	98	
	PCR primer 2	ATTAACTAAACTAATCTTAAACTCCTAACCT		
	TaqMan probe	6-FAM-CCTACCTTAACCTCCC-MGBNFQ		
PyroMeth	PCR primer 1	Biotin-GAAAGAGGGAAAGGTTTTTT	301	−217 to +84
	PCR primer 2	CCATACTAAAAACTCTAAACCCCATC		
	Seq primer 1	CGAACCTTATAAAAATAATC		−21 to −41
	Seq primer 2	GTAGTTTTYGTTATTAGTGA		+15 to +34
	Seq primer 3	CGAACCTTATAAAAATAATC		−21 to −39
MALDI-MS	PCR primer 1	GTGACTCAGCACTGG(GTGATTTAGTATTGG)	144	−70 to +74
	PCR primer 2	AGCTCTGAGCCCCATC(AACTCTAAACCCCATC)		
	Miniseq primer (−22 CpG)	GGGATTATTTTTATAAGGTT		−42 to −23
	Miniseq primer (+8 CpG)	GYGAGGTTTTYGTTGGAGTTT		−14 to +7
	Miniseq primer (+14 CpG)	TACTCACTAATAACRAAAACTAC		+37 to +15
	Miniseq primer (+38 CpG)	GTAGTTTTYGTTATTAGTGAGTA		+15 to +37

Table S1. *Cont.*

Methylation method	Oligo name	Sequence 5'–3'	Size of PCR product (bp)	Position in GSTP1 promoter (related to transcription start site, TSS)
	Miniseq primer (+47 CpG)	ATTAGTGAGTAYGYGYGGTT		+27 to +46
	Miniseq primer (+55 CpG)	AACTCTAAACCCCATCCCC		+56 to +74
Cloning	PCR primer 1	ATTTGGGAAAGAGGGAAAGGT	267	−223 to +81
	PCR primer 2	TACTAAAAACTCTAAACCCCATC		

Table 2. The percentage of methylation for each of the samples analyzed. For PyroMeth and MALDI-MS the average of the analyzed CpGs were used.

Sample ID	MethyLight A [1]	MethyLight B [2]	PyroMeth MethyLight A CpGs [3]	PyroMeth MethyLight B CpGs [4]	PyroMeth MALDI-MS CpGs [5]	PyroMeth all analyzed CpGs [6]	MALDI-MS MethyLight A CpGs [7]	MALDI-MS MethyLight B CpGs [8]	MALDI-MS all analyzed CpGs [9]
Sample01	6.7	29.7	N/A	46.1	N/A	38.6	62.2	73.5	54.3
Sample02	0.0	0.0	8.9	9.8	7.2	7.2	18.4	19.3	15.8
Sample03	0.0	0.0	24.8	21.1	25.0	21.3	43.5	39.1	40.9
Sample04	0.0	0.0	28.8	20.8	26.3	19.8	15.9	14.0	13.7
Sample05	0.0	0.0	3.4	4.2	2.9	3.5	4.0	3.5	3.7
Sample06	0.0	0.0	2.4	3.2	2.3	2.8	4.1	2.9	3.7
Sample07	8.8	44.6	45.0	41.4	46.2	40.7	37.2	31.1	34.5
Sample08	0.0	0.0	N/A	N/A	N/A	N/A	4.8	4.4	4.2
Sample09	0.0	0.0	18.8	19.1	22.1	16.3	25.4	25.9	21.7
Sample10	0.0	0.0	N/A	N/A	N/A	N/A	17.5	18.7	15.2
Sample11	2.7	13.0	53.9	54.6	52.8	52.6	49.9	52.0	45.2
Sample12	0.0	9.3	27.9	34.3	25.0	24.8	24.1	33.4	20.3
Sample13	0.0	0.0	2.8	4.0	2.6	3.2	3.5	2.3	3.2
Sample14	0.0	0.0	17.2	13.3	16.1	13.3	28.9	31.8	27.5
Sample15	2.1	18.1	38.2	42.0	36.5	33.8	48.6	62.2	41.1
Sample16	3.4	23.4	24.9	22.6	19.5	23.4	23.2	25.5	19.5
Sample17	3.9	16.7	17.3	24.1	17.2	21.7	39.0	36.3	36.0

Table S2. *Cont.*

Sample ID	MethyLight A [1]	MethyLight B [2]	PyroMeth MethyLight A CpGs [3]	PyroMeth MethyLight B CpGs [4]	PyroMeth MALDI-MS CpGs [5]	PyroMeth all analyzed CpGs [6]	MALDI-MS MethyLight A CpGs [7]	MALDI-MS MethyLight B CpGs [8]	MALDI-MS all analyzed CpGs [9]
Sample18	0.0	0.0	5.2	7.1	4.9	5.9	6.8	5.7	5.8
Sample19	0.0	0.0	2.9	2.9	3.1	3.0	3.8	3.0	3.4
Sample20	7.1	31.2	21.3	23.6	19.7	20.3	39.4	36.2	36.8
Sample21	7.0	35.6	N/A	N/A	N/A	N/A	45.7	45.9	39.6
Sample22	0.0	0.0	N/A	N/A	N/A	N/A	5.0	4.5	4.5
Sample23	0.0	0.0	N/A	N/A	N/A	N/A	N/A	N/A	N/A
Sample24	0.0	0.0	13.8	18.0	9.0	13.1	10.8	12.5	9.4
Sample25	0.0	0.0	8.6	9.1	8.6	7.1	27.4	26.9	24.0
Sample26	4.6	15.1	65.0	54.5	62.3	53.5	43.2	40.0	39.3
Sample27	N/A	0.0	29.4	33.4	30.9	27.3	38.7	34.0	35.0
Sample28	N/A	0.0	N/A	N/A	N/A	N/A	N/A	N/A	N/A
Sample29	0.0	0.0	3.1	5.7	3.2	4.3	8.6	10.5	7.1
Sample30	0.0	0.0	4.4	5.0	4.1	4.4	3.5	2.9	3.0
Sample31	0.0	0.0	2.9	5.6	3.0	4.3	4.3	3.8	3.8
Sample32	0.0	0.0	N/A	N/A	N/A	N/A	N/A	N/A	N/A
Sample33	0.0	0.0	3.3	6.1	3.4	4.6	4.2	3.2	3.7
Sample34	3.1	18.0	32.8	34.6	33.6	32.2	34.1	35.0	32.2
Sample35	0.0	19.6	48.8	46.0	48.6	43.5	34.4	36.4	33.8
Sample36	0.2	0.0	15.0	13.3	14.8	12.0	16.4	18.7	14.2
Sample37	0.0	0.0	4.3	3.9	3.8	4.0	1.6	2.4	1.4
Sample38	3.4	16.4	53.6	55.0	53.6	53.0	18.3	21.1	16.6
Sample39	N/A	N/A	3.9	8.7	4.1	7.3	8.5	8.5	7.2
Sample40	0.0	28.9	27.2	29.7	25.0	25.6	37.6	34.9	33.2
Sample41	3.3	7.4	72.0	53.8	69.1	49.4	45.2	55.9	43.2
Sample42	4.3	24.7	16.3	16.6	17.2	14.8	34.0	29.2	30.5
Sample43	N/A	0.0	N/A	7.3	N/A	6.4	22.2	24.6	19.1
Sample44	N/A	0.0	3.1	3.7	3.2	3.8	3.8	3.2	3.5
Sample45	0.0	18.8	43.3	56.1	43.2	50.3	51.8	54.3	47.1

Table S2. *Cont.*

Sample ID	MethyLight A [1]	MethyLight B [2]	PyroMeth MethyLight A CpGs [3]	PyroMeth MethyLight B CpGs [4]	PyroMeth MALDI-MS CpGs [5]	PyroMeth all analyzed CpGs [6]	MALDI-MS MethyLight A CpGs [7]	MALDI-MS MethyLight B CpGs [8]	MALDI-MS all analyzed CpGs [9]
Sample46	0.0	0.0	5.9	8.4	5.7	6.7	12.2	10.7	10.3
Sample47	N/A	0.0	N/A	N/A	N/A	N/A	N/A	N/A	N/A
Sample48	0.0	0.0	3.1	5.5	3.3	4.4	4.5	4.2	3.8
Sample49	0.0	0.0	5.4	8.1	5.9	5.8	4.8	4.3	4.2
Sample50	0.0	0.0	2.9	5.7	2.9	4.0	3.8	3.3	3.7
Sample51	0.0	0.0	36.1	32.2	38.1	27.5	27.4	28.7	32.3
Sample52	0.0	0.0	8.7	9.1	8.1	8.1	N/A	N/A	N/A
Sample53	0.0	0.0	N/A	N/A	N/A	N/A	1.4	1.7	1.2
Sample54	0.0	0.0	N/A	N/A	N/A	N/A	5.6	5.0	5.2
Sample55	0.0	0.0	31.6	22.9	32.3	22.8	33.8	32.9	29.2
Sample56	0.0	0.0	25.9	23.8	22.6	20.8	30.9	35.8	26.5
Sample57	0.0	3.0	36.5	42.5	32.8	29.6	39.3	47.2	32.8
Sample58	0.0	0.0	3.3	6.8	3.4	4.9	3.3	2.8	2.9
Sample59	0.0	0.0	N/A	N/A	N/A	N/A	N/A	N/A	N/A
Sample60	0.0	0.0	N/A	N/A	N/A	N/A	11.8	12.9	10.1
Sample61	0.0	0.0	2.8	4.7	2.8	3.3	3.1	2.7	3.1
Sample62	0.0	0.0	N/A	N/A	N/A	N/A	4.3	3.3	4.2
Sample63	0.0	0.0	30.3	32.2	28.7	28.1	43.7	49.1	38.8
Sample64	N/A	N/A	16.4	16.6	22.0	15.2	27.3	27.5	25.2
Sample65	0.0	0.0	18.9	18.1	17.5	18.2	25.8	31.9	22.1
Sample66	0.0	0.0	15.9	17.7	13.7	15.7	29.2	28.7	25.3
Sample67	0.0	0.0	44.6	42.9	44.6	39.2	44.9	39.2	41.7
Sample68	0.0	0.0	15.8	16.0	16.4	12.6	20.4	23.1	17.5
Sample69	1.4	0.0	N/A	N/A	N/A	N/A	N/A	N/A	N/A
Sample70	0.0	0.0	4.5	7.8	5.1	5.7	4.1	3.1	3.6
Sample71	0.0	0.0	7.8	11.0	8.1	8.8	12.5	13.5	10.7
Sample72	0.0	0.0	14.2	15.6	13.4	12.5	20.4	26.3	17.3
Sample73	0.0	0.0	3.4	4.6	3.2	3.7	5.9	4.9	5.3

Table S2. *Cont.*

Sample ID	MethyLight A [1]	MethyLight B [2]	PyroMeth MethyLight A CpGs [3]	PyroMeth MethyLight B CpGs [4]	PyroMeth MALDI-MS CpGs [5]	PyroMeth all analyzed CpGs [6]	MALDI-MS MethyLight A CpGs [7]	MALDI-MS MethyLight B CpGs [8]	MALDI-MS all analyzed CpGs [9]
Sample74	0.0	0.0	3.8	4.5	4.0	4.0	6.2	5.0	5.5
Sample75	0.0	12.4	50.4	52.2	48.7	47.4	60.0	57.1	55.7
Sample76	0.0	39.2	34.3	35.3	31.0	30.0	45.0	44.8	38.9
Sample77	0.0	0.0	2.7	N/A	1.9	2.6	15.0	12.1	13.1
Sample78	0.0	2.0	28.2	29.6	27.4	26.4	N/A	N/A	N/A
Sample79	0.0	0.0	4.5	4.5	4.1	4.1	4.1	3.7	3.9
Sample80	1.2	19.8	N/A	N/A	N/A	N/A	63.9	69.7	58.2
Sample81	0.0	0.0	3.0	5.1	2.6	4.0	4.4	3.1	4.1
Sample82	0.0	14.7	21.0	23.7	23.7	21.3	34.2	32.5	30.3
Sample83	0.0	0.0	13.5	10.7	10.7	10.4	10.4	9.7	9.1
Sample84	N/A	N/A	20.7	23.1	18.1	16.1	23.9	34.2	20.2
Sample85	0.0	0.0	N/A	N/A	N/A	N/A	N/A	N/A	N/A
Sample86	0.0	0.0	18.9	22.1	20.0	18.3	20.7	23.3	17.7
Sample87	0.0	0.0	2.8		1.5	2.3	4.2	4.0	3.8
Sample88	0.0	0.0	7.0	7.9	5.8	6.6	13.0	12.2	11.4

[1] MethyLight A CpG pos.: −22, −15, −13, +8, +11, +14, +38, +40, +42 and +47; [2] MethyLight B CpG pos.: −77, −74, −71, −22, −15, −13, +8, +11 and +14; [3] PyroMeth average of CpG pos. covered by the MethyLight A primers and probe: −22, −15, −13, +8, +11, +14, +38, +40, +42 and +47; [4] PyroMeth average of CpG pos. covered by the MethyLight B primers and probe: −77, −74, −71, −22, −15, −13, +8, +11 and +14; [5] PyroMeth average of CpG pos. also analyzed by MALDI-MS: −22, +8, +14, +38, +47 and +55; [6] PyroMeth average of all CpGs analyzed: pos. −77, −74, −71, −48, −43, −22, −15, −13, +8, +11, +14, +38, +40, +42, +47 and +55; [7] MALDI-MS average of CpG pos. covered by the MethyLight A primers and probe: −22, −15, −13, +8, +11, +14, +38, +40, +42 and +47; [8] MALDI-MS average of CpG pos. covered by the MethyLight B primers and probe: −77, −74, −71, −22, −15, −13, +8, +11 and +14; [9] MALDI-MS average of all CpGs analyzed: pos. −22, +8 and +14, +38, +47 and +55; N/A—not analyzed.

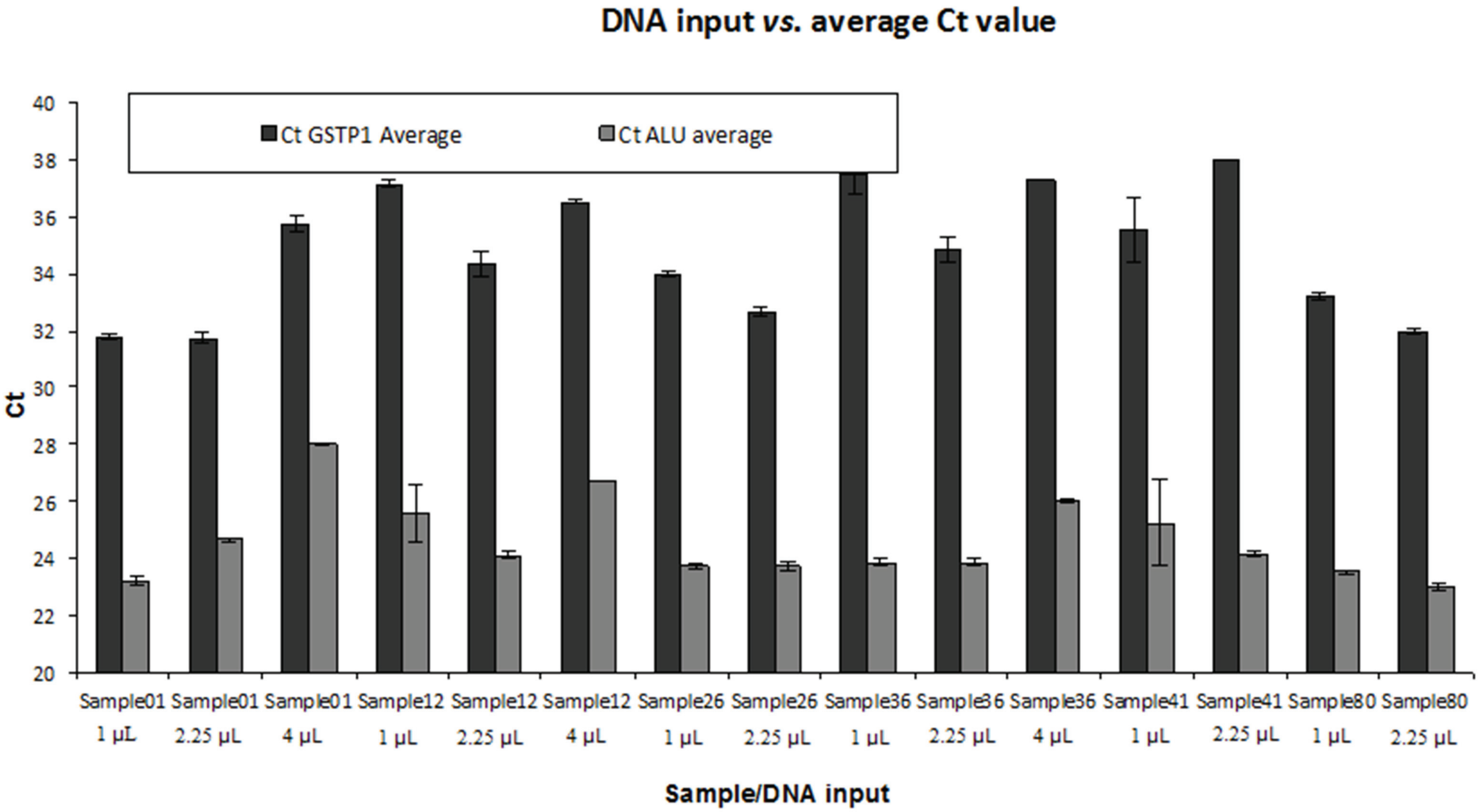

Figure S1. Increasing the input concentrations of bisulphite treated DNA in the MethyLight B reaction did not lower the Ct values for sample 01, 12, 26 36, 41 or 80. This suggests that the amplification the lack of DNA methylation detection was not caused of lack of input DNA.

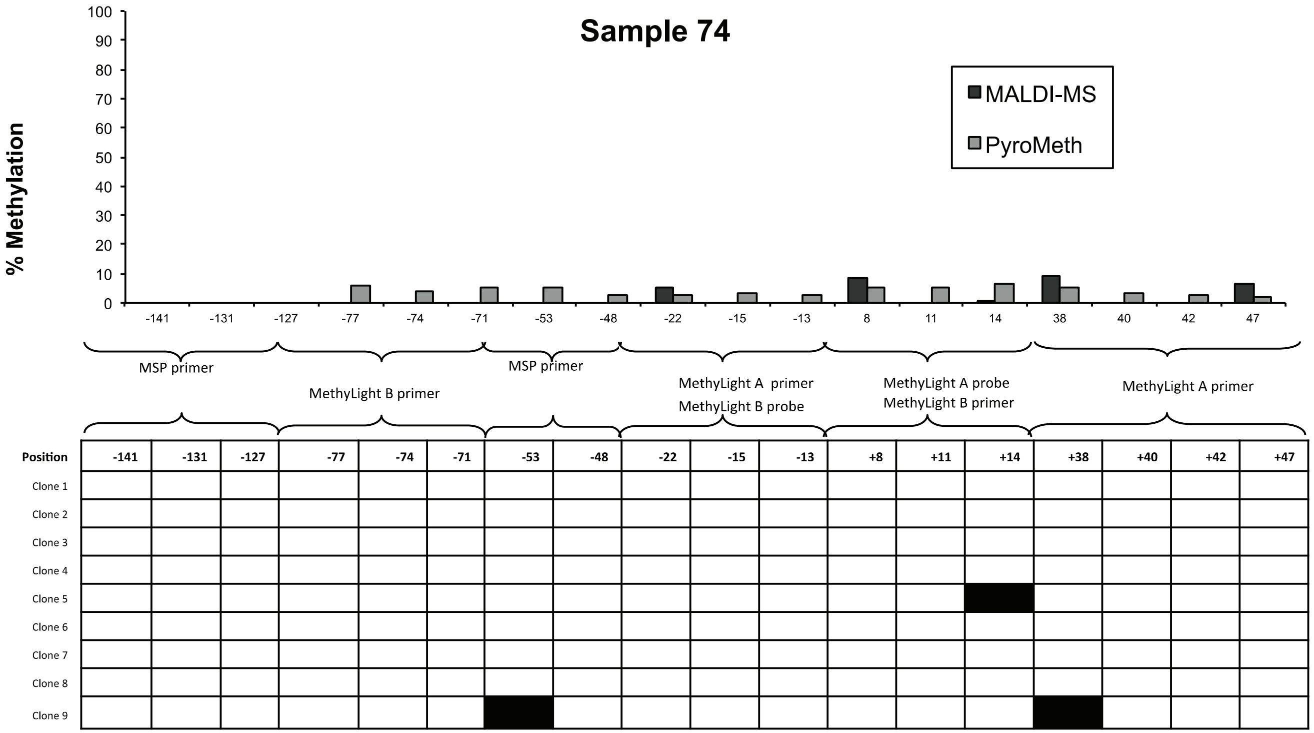

**PyroMeth** average % methylation		**MALDI-MS** average % methylation		**MethyLight A**	**MethyLight B**	**MSP**
CpGs covered by MethyLight A assay	CpGs covered by MethyLight B assay	CpGs covered by MethyLight A assay	CpGs covered by MethyLight B assay	Primers and probe cover CpGs -22, -15, -13, +8, +11, +14, +38, +40, +42, +47	Primers and probe cover CpGs -77, -74, -71, -22, -15, -13, +8, +11, +14	Primers covering CpGs -141, -131, -127, -53 and -48
**3.8**	**4.5**	**6.2**	**5.0**	**0**	**0**	**unmethylated**

Methylated Unmethylated Unknown

(**A**)

Figure S2. (**A**) Results from cloning and sequencing of the *GSTP1* promoter for sample 74; (**B**) Results from cloning and sequencing of the *GSTP1* promoter for sample 13; (**C**) Results from cloning and sequencing of the *GSTP1* promoter for sample 35; (**D**) Results from cloning and sequencing of the *GSTP1* promoter for sample 15; (**E**) Results from cloning and sequencing of the *GSTP1* promoter for sample 12; (**F**) Results from cloning and sequencing of the *GSTP1* promoter for sample 03.

Figure S2. *Cont.*

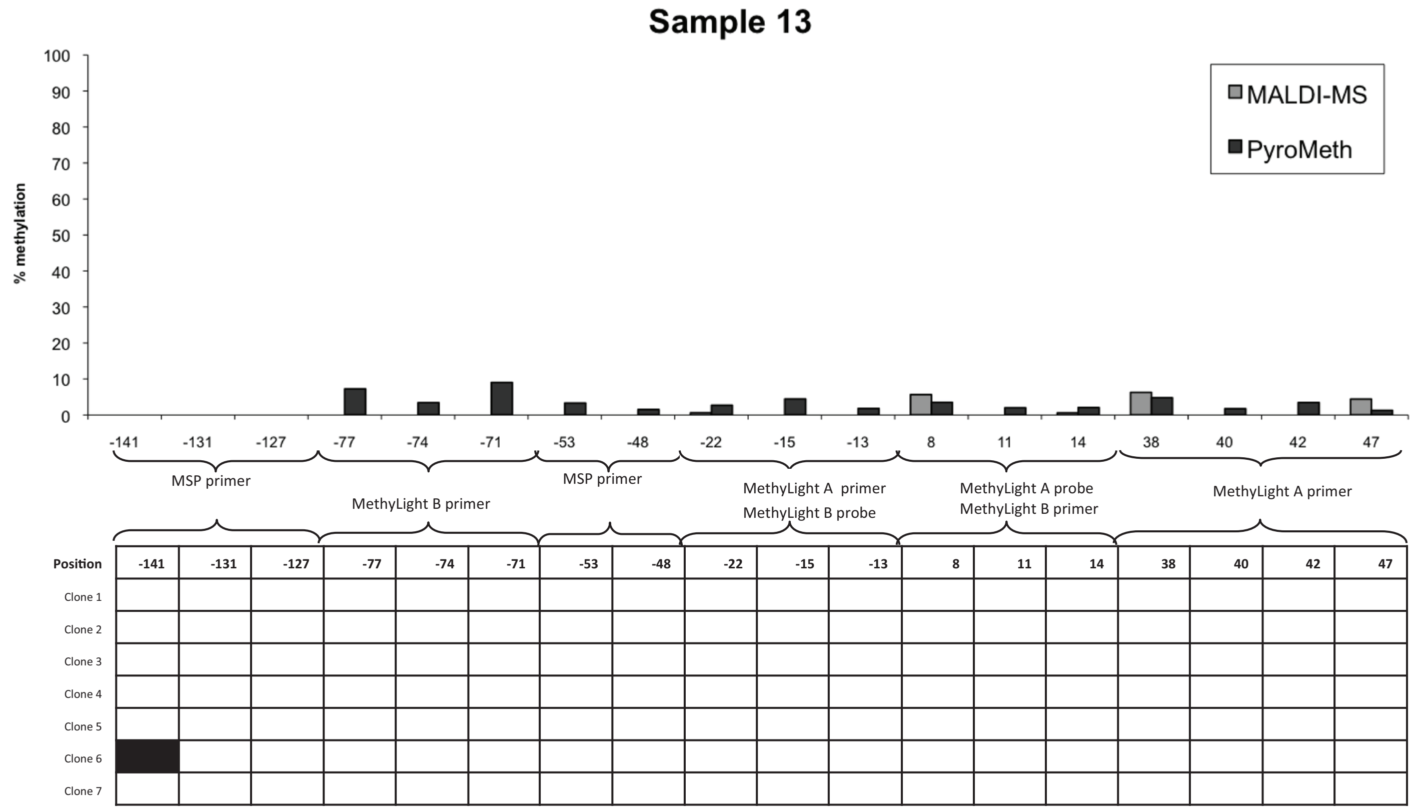

**PyroMeth** average % methylation		**MALDI-MS** average % methylation		**MethyLight A**	**MethyLight B**	**MSP**
CpGs covered by MethyLight A assay	CpGs covered by MethyLight B assay	CpGs covered by MethyLight A assay	CpGs covered by MethyLight B assay	Primers and probe cover CpGs -22, -15, -13, +8, +11, +14, +38, +40, +42, +47	Primers and probe cover CpGs -77, -74, -71, -22, -15, -13, +8, +11, +14	Primers covering CpGs -141, -131, -127, -53 and -48
**2.8**	**4.0**	**3.5**	**2.3**	**0**	**0**	**unmethylated**

■ Methylated □ Unmethylated ▩ Unknown

(B)

Figure S2. *Cont.*

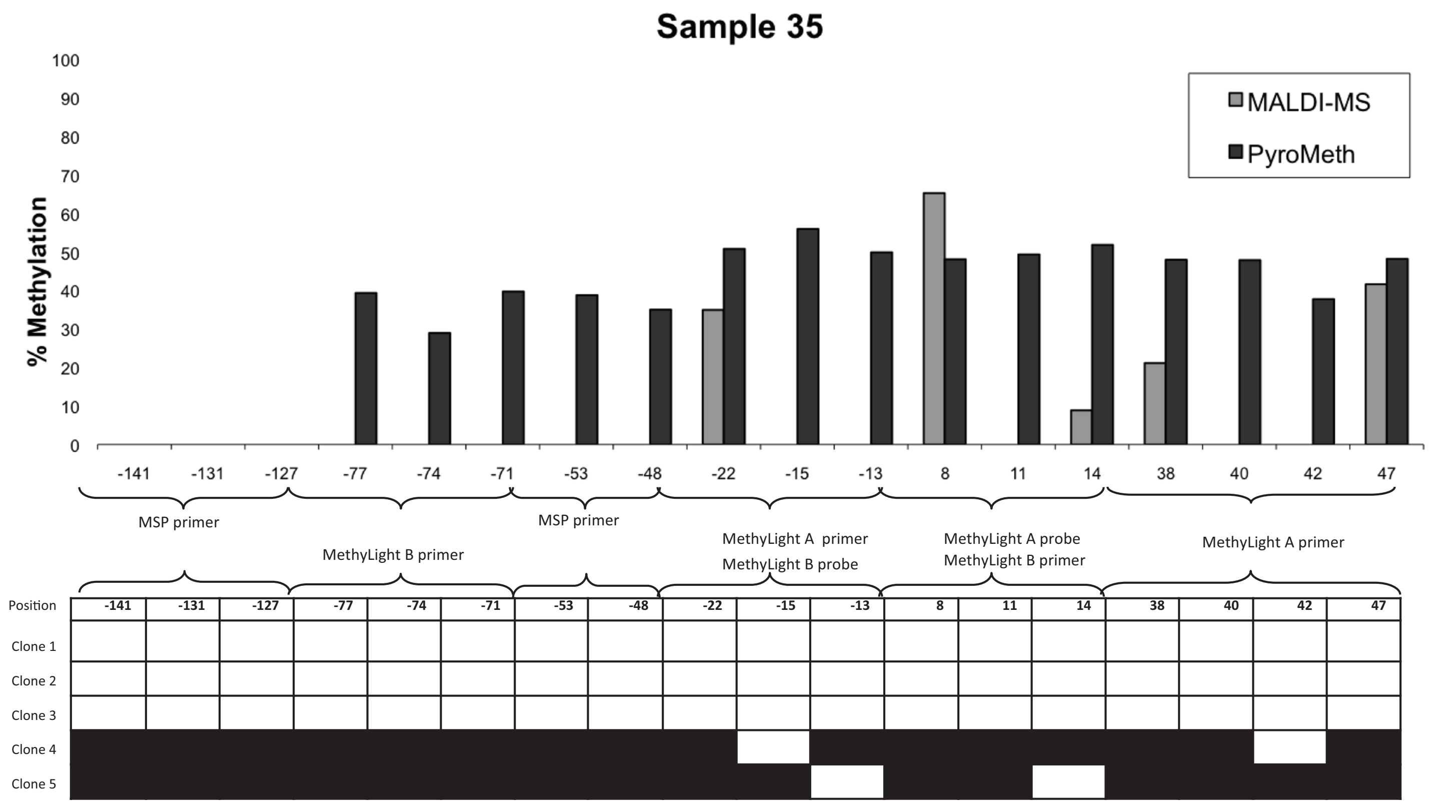

**PyroMeth** average % methylation		**MALDI-MS** average % methylation		**MethyLight A**	**MethyLight B**	**MSP**
CpGs covered by MethyLight A assay	CpGs covered by MethyLight B assay	CpGs covered by MethyLight A assay	CpGs covered by MethyLight B assay	Primers and probe cover CpGs -22, -15, -13, +8, +11, +14, +38, +40, +42, +47	Primers and probe cover CpGs -77, -74, -71, -22, -15, -13, +8, +11, +14	Primers covering CpGs -141, -131, -127, -53 and -48
**48.8**	**46.0**	**34.4**	**36.3**	**0**	**1.6**	**methylated**

■ Methylated □ Unmethylated ■ Unknown

(C)

Figure S2. *Cont.*

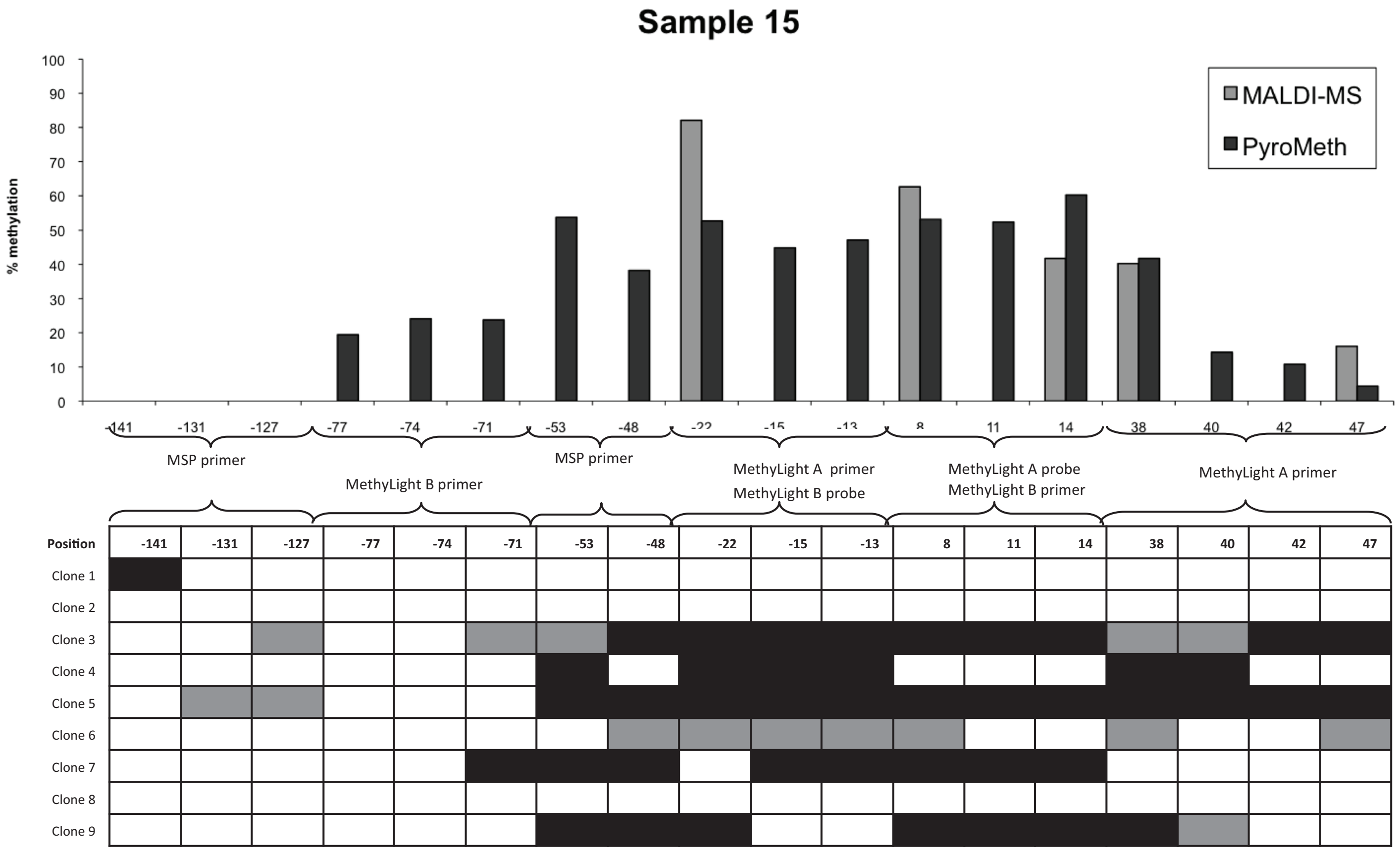

**PyroMeth** average % methylation		**MALDI-MS** average % methylation		**MethyLight A**	**MethyLight B**	**MSP**
CpGs covered by MethyLight A assay	CpGs covered by MethyLight B assay	CpGs covered by MethyLight A assay	CpGs covered by MethyLight B assay	Primers and probe cover CpGs -22, -15, -13, +8, +11, +14, +38, +40, +42, +47	Primers and probe cover CpGs -77, -74, -71, -22, -15, -13, +8, +11, +14	Primers covering CpGs -141, -131, -127, -53 and -48
**38.2**	**42.0**	**48.6**	**62.6**	**2.1**	**18.1**	**unmethylated**

■ Methylated □ Unmethylated ▒ Unknown

(D)

Figure S2. *Cont.*

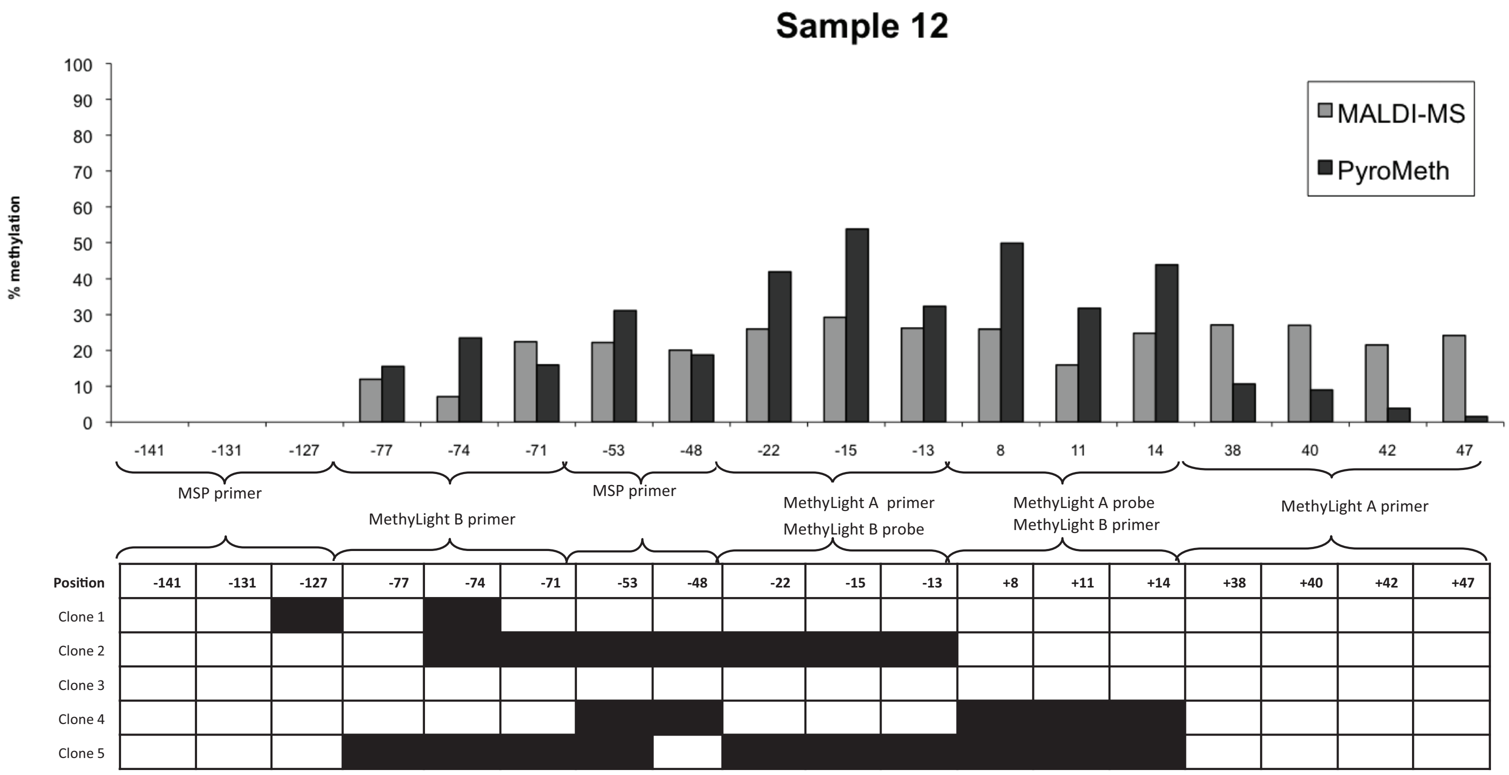

**PyroMeth** average % methylation		**MALDI-MS** average % methylation		**MethyLight A**	**MethyLight B**	**MSP**
CpGs covered by MethyLight A assay	CpGs covered by MethyLight B assay	CpGs covered by MethyLight A assay	CpGs covered by MethyLight B assay	Primers and probe cover CpGs -22, -15, -13, +8, +11, +14, +38, +40, +42, +47	Primers and probe cover CpGs -77, -74, -71, -22, -15, -13, +8, +11, +14	Primers covering CpGs -141, -131, -127, -53 and -48
**27.9**	**34.3**	**24.1**	**33.4**	**0**	**9.3**	**unmethylated**

Methylated Unmethylated Unknown

(E)

Figure S2. *Cont.*

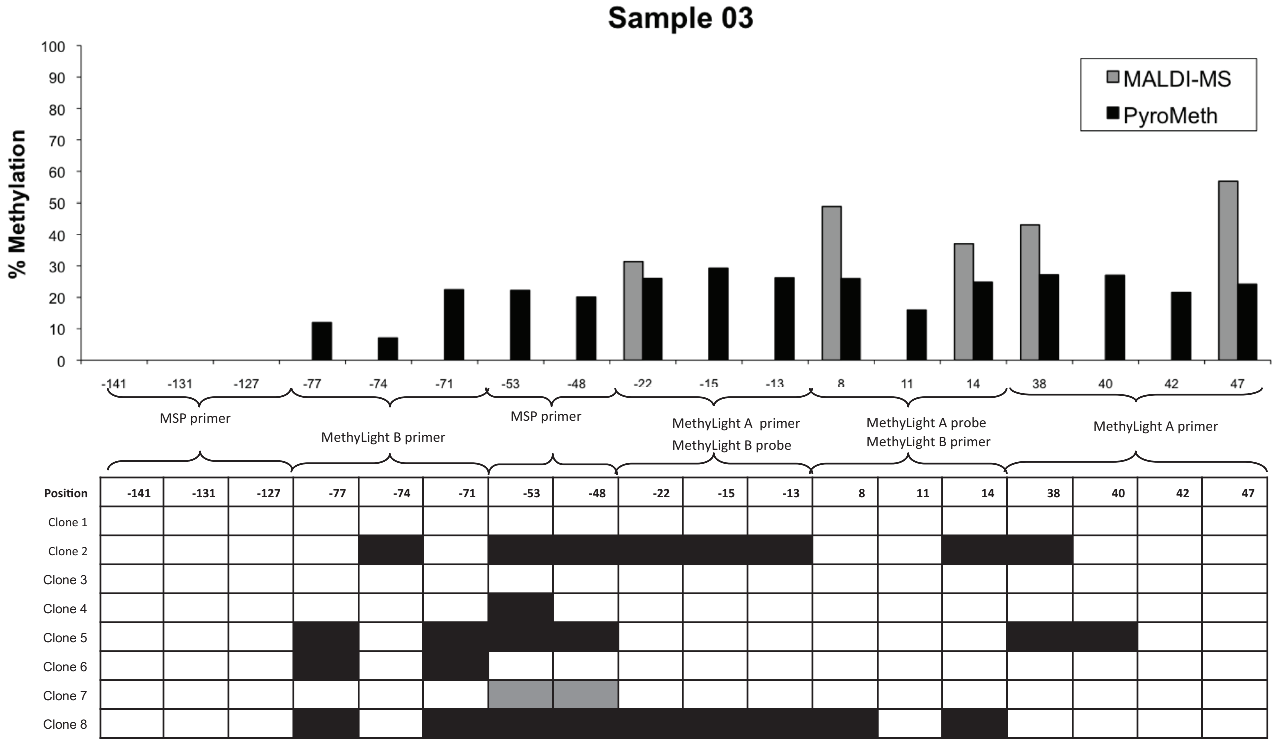

**PyroMeth** average % methylation		**MALDI-MS** average % methylation		**MethyLight A**	**MethyLight B**	**MSP**
CpGs covered by MethyLight A assay	CpGs covered by MethyLight B assay	CpGs covered by MethyLight A assay	CpGs covered by MethyLight B assay	Primers and probe cover CpGs -22, -15, -13, +8, +11, +14, +38, +40, +42, +47	Primers and probe cover CpGs -77, -74, -71, -22, -15, -13, +8, +11, +14	Primers covering CpGs -141, -131, -127, -53 and -48
**24.8**	**21.1**	**43.5**	**39.1**	**0**	**0**	**unmethylated**

Methylated Unmethylated Unknown

(F)

© 2015 by the authors; licensee MDPI, Basel, Switzerland. This article is an open access article distributed under the terms and conditions of the Creative Commons Attribution license (http://creativecommons.org/licenses/by/4.0/).
